# Detection of misfolded protein aggregates from a clinical perspective

**Published:** 2016-03-22

**Authors:** Øyvind Strømland, Martin Jakubec, Samuel Furse, Øyvind Halskau

**Affiliations:** Department of Molecular Biology, University of Bergen, Bergen, Norway

**Keywords:** protein misfolding, Alzheimer's, Parkinson's, neurodegenerative disease, oligomer toxicity, membrane porosity, lipid, antibody, lectin, mass spectrometry

## Abstract

Neurodegenerative Protein Misfolding Diseases (PMDs), such as Alzheimer’s (AD), Parkinson’s (PD) and prion diseases, are generally difficult to diagnose before irreversible damage to the central nervous system damage has occurred. Detection of the misfolded proteins that ultimately lead to these conditions offers a means for providing early detection and diagnosis of this class of disease. In this review, we discuss recent developments surrounding protein misfolding diseases with emphasis on the cytotoxic oligomers implicated in their aetiology. We also discuss the relationship of misfolded proteins with biological membranes. Finally, we discuss how far techniques for providing early diagnoses for PMDs have advanced and describe promising clinical approaches. We conclude that antibodies with specificity towards oligomeric species of AD and PD and lectins with specificity for particular glycosylation, show promise. However, it is not clear which approach may yield a reliable clinical test first.

**Relevance for patients:** Individuals suffering from protein misfolding diseases will likely benefit form earlier, less- or even non-invasive diagnosis techniques. The current state and possible future directions for these are subject of this review.

## 1. Introduction

The oligomerization and then fibrillation of misfolded proteins is a common feature of a large group of diseases referred to as protein misfolding diseases (PMDs). A subset of these diseases is known as neurodegenerative because they cause irreversible damage to the central nervous system (CNS). Several different proteins may precipitate the clinical symptoms of these conditions, several parts of the CNS may be damaged and the mechanism of the condition varies (Table 1). The best known examples of neurodegenerative PMDs include Parkinson’s [1,2] and Alzheimer’s [2-4] diseases (PD and AD, respectively). Prionic diseases are less common and less predictable than the others but their immediate clinical impact can be more dramatic [5,6]. There are also a number of hereditary conditions also counted among the neurodegenerative PMDs, the most well- known of these being Huntington’s Disease (HD) [7]. While the exact nature and relevance of protein misfolding is sometimes debated, for instance the relevance and nature of Huntingtin (Htt) aggregation in HD [8,9], there is agreement that misfolded, mis-aggregated or wrongly processed proteins are the unifying feature of these conditions [10]. Full recovery has not yet been observed in any patient after damage to nerve tissue has begun. Clear and early diagnosis of these conditions is therefore essential for informing sufferers about their condition, managing the condition where this is possible and giving appropriate palliative care. Theoretically, early diagnosis might help guide choice of treatment in cases where effective options are available.

**Table 1.** Neurodegenerative protein misfolding diseases

Protein Misfolding Disease	Aggregating protein(s)	Aetiology	Clinical Manifestation	Pathogenic mechanism
AD	Aβ, Tau [128]	Acquired; age, gene variants increase risk, see also familial forms	Dementia, language difficulties, executive dysfunction, depression, hallucinations, delusions, agitation, apathy, disinhibition [128]	Depositions of Aβ plaques and Tau tangles observed. Selective loss of cholinergic neurones, loss of synapses and neurones in the cerebral cortex, atrophy of frontal cortex cingulate gyrus, temporal lobe and parietal lobe [129]
Cerebral amyloid angiopathy	Aβ, BRI2, Cystatin C, gelsolin, PrPSc, Transthyrin [130]	Acquired; age, familial factors and familial subtypes types identified	Cerebral haemorrhage, ischemic lesions, progressive dementia [130]	Progressive deposition of amyloid protein in cerebral blood vessel walls leading to degenerative vascular changes [130]
PD	α-Syn, Tau [131,132]	Acquired; head trauma, specific gene variants known to increase risk	REM sleep behaviour disorder, Excessive daytime sleepiness, hyposmia, depression, bradykinesia, rigidity, tremors, mild cognitive impairment, dyskinesia, dysphagia, postural instability, freezing of gait, orthostatic hypotension [131]	Manifestation of Lewy bodies enriched in α-Syn. Loss of dopaminergic neurons in the substantia nigra, Neuroinflammation with reactive gliosis and microgliosis [131]
Frontotemporal lobar degeneration	Tau, TDP-43, FUS, p62, ubiquitin [133]	Major genetic contributions [134]	Personality changes, behavioural disinhibition, apathy, progressive aphasia [53]	Neuronal loss, gliosis, microvacular changes of frontal lobes, anterior temporal lobes, anterior cingulate cortex and insular cortex [133]
Huntington’s disease	Htt [135]	Congenital, monogenic	Mild psychotic and behavioural symptoms, progressive chorea, rigidity, dementia, dystonia, bradykinesia [135]	Gross striatal atrophy, neuronal loss in neocortex, cerebellum, hippocampus, substantia nigra, and brainstem nuclei [135]
Familial British dementia, and Familial Danish dementia	BRI2 [136]	Congenital, monogenic	Progressive cognitive impairment, spastic tetraparesis, cerebellar ataxa [137]	Amyloid angiopathy and neurofibrillary tangles (NFTs) in the hippocampus [136]
CADASIL, Cerebral autosomal dominant arteriopathy with subcortical infarcts and leukoencephalopathy	NOTCH3 [138]	Congenital, monogenic [139]	Mood disturbances, apathy, subcortical ischemic events, migraine with aura, cognitive impairment [138]	Degeneration of smooth muscle cells in blood vessels [138]
Alexander disease	GFAP [140]	Sporadic; gene variants increase risk	Macrocephaly, frontal leukodystrophy, palatal tremors, dysphagia, cognitive delays, seizures [140]	Demyelination, Rosenthal fibres in astrocytes [141]
Familial encephalopathy with neuroserpin inclusion bodies	Neuroserpin [142]	Congenital, monogenic [143]	Dementia, epileptic, seizures, progressive myoclonus, dysarthria [142]	Poorly understood, encephalopathy with neuroserpin inclusion bodies [142]
Kuru	PrP [144]	Acquired; transmitted	Cerebellar ataxia, choreifrom, athetoid movements, nystagmus, dysphasia [144]	Spongiform change, neuronal loss, astrocytic microgliosis, kuru plaques [144]
Creutzfeldt-Jakob disease	PrP [145]	Acquired; transmitted	Dementia, myoclonus, visual or cerebellar disturbance, akinetic mutism, pyramidal or extrapyramidal signs [146]	Spongiform change, neuronal loss, gliosis [145]
Gerstmann-Straussler-Scheinker syndrome	PrP [144]	Major genetic contributions [147]	Cerebellar ataxia, gait abnormalities, dementia, dysarthria, ocular dysmetria, myoclonus, spastic paraparesis, parkinsonism, hyporeflexia or areflexia in lower extremities [144]	Amyloid plaques, severe to absent spongiform changes, neuronal loss, astrocyte microgliosis, variable neurofibrillary tangles [144]
Fatal familial insomnia	PrP [144]	Congenital, monogenic [148]	Insomnia, myoclonus, ataxia, dysarthria, dysphagia, pyramidal signs, autonomic hyperactivation [144]	Neuronal loss, astrogliosis, hypometabolism in the thalamus and cingulate cortex [144]
Progressive supranuclear palsy	Tau [149]	Acquired; head trauma [150]	Progressive axial rigidity, vertical gaze palsy, dysarthria, dysphagia [149]	Neuronal loss, gliosis, neurofibrillary tangles affecting brainstem, basal ganglia, diencephalon [149]
Chronic traumatic encephalopathy	Tau, TDP-43 [151]	Acquired; head trauma [152]	Learning and memory impairment, anterograde amnesia, executive dysfunction, depression, apathy, irritability, suicidality, loss of impulse control, dementia, PD, dysarthria [151]	Atrophy of frontal and temporal cortices and medial temporal lobe, atrophy of the thalamus, hypothalamus and mammillary bodies. Thinning of the corpus callosum, pallor of the substantia nigra and locus coeruleus, cavum septum pellucidum [151]
Lytico-Bodig disease	Tau [153]	Acquired	Global dementia, progressive aphasia, gaze palsy, parkinsonism, progressive supranuclear palsy [153]	Poorly understood, neurofibrillary tangles are found in the brain [153]
Meningioangiomatosis	Tau [154]	Acquired	Epileptic seizures, haemorrhagic stroke, anginoma, status epilepticus, generalized tonic–clonic seizures [155]	Focal lesion of the leptomeninges and underlying cerebral cortex [155]
Neuronal Ceroid Lipofuscinosis	ATP synthase subunit c, saposin A, saposin D [156]	Congenital, monogenic, subtypes exists [157]	Hypotonia, myoclonic jerks, generalized epileptic seizures, developmental regression, optic atrophy, macular degeneration, spastic tetraplegia, blindness, severe and constant microcephaly, and pharmaco-resistant epileptic seizures, myoclonia, ataxia, extrapyramidal signs [156]	Cerebellar and cortical atrophy, loss of pyramidal neurons and Purkinje cells, reactive astrogliosis [156]
Argyrophilic grain disease	Tau [158]	Acquired; old age [158]	Cognitive decline, dementia, mood imbalance, personality changes, behavioural abnormalities [158]	Argyrophilic grains in trans entorhinal cortex, entorhinal cortex, hippocampus, presubiculum, temporal cortex, orbitofrontal cortex, insular cortex, and amygdala[158]

The desire for both prompt diagnosis and improved medical treatments has thus encouraged research into misfolded protein aggregates and their relative PMDs. Diligent research has identified and characterised many of the individual proteins involved in these conditions. For example, β-amyloid (Aβ) [11] and Tau in AD [12], *α*-Synuclein ( *α*-Syn) in PD [13,14] and Htt in HD [7] are now largely accepted to have key roles in these diseases (For other protein involvement in given diseases, see Table 1). Despite this, tests for diagnostic compounds (biomarkers) are not in routine use for identifying any of the non-hereditary PMDs. For instance, when assessing patients for AD, clinicians have to rely on an imaging or visual data regarding symptoms and standardized tests that are sometimes combined with MRI [7]. Biopsies for detecting changes in the CNS are considered invasive procedures that are generally unsuitable for elderly patients, and thus they are usually only used for verification of the diagnosis post mortem [15]. Importantly, tissue damage precedes the formation of the characteristic insoluble fibrils that are detected in brains of AD sufferers. Such fibrils are not cytotoxic and their formation correlates only poorly with disease progression [16]. This suggests that other agents, such as oligomers of the same misfolded proteins as the fibrils, are responsible.

The common features of neurodegenerative PMDs, in which only one or two proteins appear to be defective, or at least the processing of which is defective, is an attractive target for translational research aiming to detect the condition in its early stages. One possible diagnostic tool is therefore a detection system for the activity of proteases that are also involved in the progression of the disease, such as caspase-8 activation in the case of PD [17] and β- and γ-secretase in AD [18,19]. One problem with this approach is that such processes do not have a unique association with the diseases in question. Another approach would be to focus on the individual misfolded proteins at an early stage, rather than the insoluble plaques and reduction in tissue volume associated with advanced stages. Sensitive tools are required for the early detection of conditions where the underlying biochemical changes may be small or difficult to resolve. For example, only minute quantities of transmissible misfolded prions are required to precipitate Creutzfeldt-Jakob Disease (CJD) in humans [6], bovine spongiform encephalopathy (BSE) and scrapie in sheep [20]. Nor is it necessarily straightforward to detect changes in protein folding, aggregation or processing in bodily fluids. Despite these challenges, methods for monitoring such changes in the relevant proteins of PMDs are of clinical interest. In this review, we explore recent advances in translational research focused on detecting misfolded proteins in the context of early pre-fibril misfolding. We discuss these advances from both a research and a clinical perspective. We conclude with a forward-looking view on possible research directions.

## 2. Protein misfolding, oligomerisation and toxicity

Proteins pass through a fundamental process called folding in order to obtain their functional structure [21-23]. Folding is usually spontaneous under physiological conditions, and occurs at rates that depend upon the protein’s size [24]. This process takes a couple of hundred milliseconds for most proteins [25]. Early steps in protein folding include the clustering of hydrophobic amino acids and the expulsion of water, the subsequent compaction of the polypeptide chain and consolidation of secondary structure. Then, a re-ordering and fine-tuning of the structural elements takes place to afford the final tertiary structure. The folding process needs to make particular intra-fold contacts both in its early and late stages. All these steps are affected by the protein’s environment. Factors that affect the outcome of a folding process and aggregation behaviour include solvent conditions, the presence or absence of cofactors and metal cations [26], chaperones or other dissolved factors, crowding from other proteins or macromolecular aggregates, spatial organization and post-translational modification (reviews [27,28]). The fact that folding is influenced by many factors is also reflected in the cell biology of the PMDs. The state of the prion protein associated with CJD is both translocated into the ER and glycosylated differently than its non-pathogenic counterparts [29,30]. Copper and zinc ions are implicated in both AD and PD, as there is evidence that they influence disease onset and progress in animal models and have been highlighted in clinical studies [31]. It is not entirely clear whether Cu^2+^ and Zn^2+^ are only involved in the misfolding of proteins or also in the aggregation of those proteins into fibrils [32].

Amyloid fibrils are widely recognized as a result of protein misfolding and have been observed both *in vitro* and *in vivo*. They are repetitive sheets in which monomers are joined by hydrogen bonds across β-strands. The long sheets are slightly twisted, with varying dimensions and crossover distances depending on the polypeptide involved. Cryo-electron microscopy has indicated that in Aβ the fibril is approximately 4 nm and 11 nm at the narrowest and widest points respectively and has a twist crossover distance that has a mean of about 100 nm. Comparative work on fibril morphology from A *β* and *α*-Syn indicates that they are similar, but with a degree of polymorphism [33-35]. A considerable range of proteins and protein fragments can form fibrils, suggesting that the barrier to formation of these states is more likely to rest with time and physico-chemical conditions than amino acid sequence.

Importantly, the extent of fibril formation does not appear to correlate with disease progression and naturally occurring mutants associated with the early onset of PMDs do not produce more fibrils [16]. For these reasons, attention has been given to the aggregates preceding fibril formation. It has been proposed recently that the toxic oligomers are, just like fibrils, a general phenomenon that forms relatively independently of protein sequence [36]. The oligomerisation of other proteins such as calcitonin [37], *α*-Syn [38], Syrian hamster prion protein [39], GAP-43 and BASP1 [40] is consistent with this. The oligomers have since been shown to display significant toxicity-related effects relative both to monomers, fibrils and proto-fibrils [41,42]. Furthermore, rates of their formation are better able to account for disease-promoting mutations [16,37,42,43]. It has also been suggested that their toxicity is linked to membrane damage through a pore-like action [44] and a range of pre-fibrillar oligomeric structures from a several proteins, including Aβ, Htt, prion proteins, and *α*-Syn, has since been investigated in this context [39,43,45,46].

## 3. Misfolded proteins and the lipid profile of the membrane

It is well established that membrane or peripheral proteins may affect membranes and *vice versa* (review [47]). The lipid-dependent, differential processing of APP to A *β* is one particularly relevant example of this [48]. Moreover, PMD proteins and notably their oligomeric states have considerable effects on membrane integrity. Furthermore, tissue deposits of amyloid fibrils contain lipids [49]. Imaging studies on prefibrillar oligomers reveal a range of structures, some of which may have a pore-like morphology (review [50]). These are referred to as amyloid pores or sometimes annular oligomers [51]. Whether such oligomers will go on to form mature amyloid fibrils exclusively is not clear, as there exists reports in which pore-like oligomers do not appear to undergo fibrillation [52]. Certain drugs can arrest fibril but not oligomer formation [53]. Even though the presence of non-fibrillar, pore-like oligomers correlates better with toxicity and disease-promoting mutants than fibrils, their properties, mechanism of action and what promotes or suppresses their formation remains poorly understood.

Two competing hypotheses that may explain the effect of oligomers on the membrane are being researched at the moment. The first suggests that oligomer toxicity is a direct result of pore formation. Examples of oligomers that may effect pore formation include those generated by islet amyloid polypeptide [54-57], poly-glutamine [58], transthyretin [59], prion protein fragment [60], A*β* [61], *β*2-microglobulin [62] and serum amyloid A [63]. Porosity was indicated by ionic flux across reconstituted membranes, which compromises cellular homeostasis and membrane potential [61]. The second hypothesis being tested at present is that the oligomers cause membrane thinning rather than leakage, through a distinct pore [42,64,65]. In this scenario, leakage through the membrane is independent of the pore-like aggregate morphologies and can take place through any area of the membrane that is sufficiently perturbed by these aggregates. Membrane thinning involves the increase of area per lipid and intercalation of polypeptides and water molecules between head groups in order to avoid energetically costly vacuums in the lateral lipid packing. This has the effect of lowering the dielectric barrier and allowing ion leakage through the membrane [10,66].

Experimental determination of leakage through a pore formation or through thinning of the membrane is not straightforward. Regardless of the particular mechanism, the oligomers convey toxicity by perturbing the integrity of the membrane. However, the membranes may in turn affect the oligomers, too. Aggregating, oligomeric peptides have been shown to have preferential binding to particular membrane components, in particular sphingolipids and cholesterol. Sphingolipids and cholesterol are found in patches termed the liquid ordered phase that are often referred to as lipid rafts [67], though controversy about this link exists [68]. Glycosphingolipids and gangliosides have affinity for the Aβ peptide in AD and *α*-Syn interacts with GM_1_ and GM_3_ gangliosides [69-74]. PrP has been associated with sphingolipid signalling platforms and bind to sphingomyelin, GalCer, GM_1_ and GM_2_ [70,75,76]. A strong interaction with sphingolipids may reflect the amount of amyloidogenic protein found in possible lipid raft areas of the extracellular leaflet of the plasma membrane [49]. Amyloidogenic proteins such as *α*-Syn also interact more strongly with anionic lipids phosphatidylglycerol and phosphatidylserine, found mainly in the cytoplasmic leaf [45,77-79]. Model systems comprising the anionic lipids cardiolipin, phosphatidic acid or phosphatidylglycerol leak more on contact with oligomers [45].

The role of cholesterol in membrane behaviour has been researched in some depth [80-88] but its role in oligomer formation and amyloidogenesis remains disputed and controversial [89]. Cholesterol has been shown to bind to Aβ proto-fibrils, but how these interactions influence oligomerization and later fibrillogenesis remains unclear [90-92]. There is evidence that cholesterol can have a stabilizing effect on membrane permeability as it reduces the leakage induced by *α*-Syn [46]. The role of cholesterol in the proteolysis of APP to give A *β* is better understood. Proteolysis of APP is inhibited by the group of cholesterol synthesis inhibitors known as statins [93,94]. In PD a depletion of cholesterol leads to a decreased level of *α*-Syn in membrane fractions in neuronal cell cultures and mouse brains [95]. Inhibition of cholesterol synthesis also reduces the levels of *α*-Syn in membranes, but the opposite applies to cholesterol supplementation in neuronal cells [96]. It has also been suggested that oxidised cholesterol accelerates aggregation of *α*-Syn [97].

This evidence may be at odds with the observation that cholesterol protects artificial membranes against oligomerinduced leakage [46] as it fails to provide a direct connection to the proposed toxicity mechanism. Polyunsaturated fatty acids may have an inhibitory role in oligomerization. The presence of docosahexaenoic acid (DHA) suppresses the toxicity of A *β* towards SH-SY5Y cells by interfering with its aggregation [98,99], and appears to have a neuro-protective role in murine models for AD [100]. However, DHA can also affect the progress of some cells through the cell cycle [101]. The notion that saturation levels of the fatty acid residues (FARs) of phospholipids in membranes play a role in modulating the rate of oligomerization agrees with measurements from model systems that indicate that saturated fatty acid residues lower the energetic barrier to aggregation [102].

Further work is required to understand the complexities of the relationship between membrane components and protein misfolding and oligomerization. It is possible that certain lipids or other membrane components may be used as diagnostic compounds for more reliable early-stage detection of neurodegenerative PMDs in combination with detection of the oligomeric proteins, should the links between lipid species and oligomerization prove robust.

## 4. Clinical detection of protein aggregates

There is no single rigorous assay for diagnosis of any PMD. The mounting evidence for the involvement of toxic oligomers in neurodegeneration confers an increasing importance on detection methods for basic and translational research, and in clinical practice. As a result, great research effort is being focused on developing clinical methods for detecting the main pathological unit of AD. At present, diagnosing AD includes a test of cognitive impairment (The Mini Mental State Exam or Folstein test), in some cases supplemented by CSF assays for phosphorylated tau and A*β*, MRI for brain volume and PET scans for Aβ plaques (or glucose metabolism) in the brain [103].

An overview of methods for clinical detection of protein aggregates is shown in Table 2. Generally, approaches for the identification of protein aggregates can be divided into three classes of method: (i) visualization of protein aggregates in biopsies, (ii) monitoring of marker peptide in bodily fluids, and (iii) visualization of protein aggregates *in vivo* using imaging techniques. Most of the methods discussed here concern Aβ peptide detection in AD, as this field has advanced the furthest. The majority of approaches rely on antibodies to confer specificity to the detection, whether it occurs in biopsies, bio-fluids or *in vivo*. A considerable number of different antibodies have been developed in the last two decades, many of which have at least some degree of specificity towards the proteins and aggregation-states involved in neurodegenerative PMDs. An overview of some of their properties is shown in Table 3a.

The visualization of amyloid plaques in samples from biopsies is a well-established means for qualitative detection of mature fibrils. There are several standard stains, such as Congo red and fluorescent thioflavins [104], as well as immunohistology stains based on antibodies [105]. There have been several recent advances in the development of fluorescent probes based on luminescent conjugated oligothiophenes (LCOs) which can be used for investigating the nature of these protein deposits [106,107]. LCOs are able to illuminate more protein deposit plaques than other fluorophores [108]. Moreover, the emission spectra of LCOs are dependent on the type of predominant peptide present. This makes it possible to distinguish e.g. AD-associated aggregates from other types of aggregates [109,110]. Another new and promising way of detecting of amyloid plaques is the discovery of photo-induced electron transfer probes that can be used to detect Aβ aggregates without the need of a washing step [111]. These recently-developed fluorescent probes represent a new opportunity in direct and sensitive identification of protein aggregates, especially in complex biological environments.

The second class of methods for the detection of proteins and their aggregates detects molecules in bio-fluid samples, avoiding the need for biopsies. These clinical methods rely on the detection of marker peptides in cerebrospinal fluid (CSF). For instance, detection of A *β* or tau protein in AD has been shown to have predictive power over which individuals will go on to develop the disease [112]. Detection of the relevant molecular species in CSF is relatively straightforward and a broad array of methods exists for its detection. It is possible to detect and quantify tau peptides in the lower ng/mL range using mass spectrometry on samples acquired directly from the CNS [12], although this not in routine clinical use yet.

A more common method is the use of enzyme-linked immuno-sorbent assays (ELISA) [113]. For example, antibodies 2G3 and 21F12 are used for the detection of C-terminal amino acids of A*β* peptides 1-40 and 1-42, respectively, in diagnosis of AD [114]. The same peptides can be detected by new electrochemical detection immuno-sensors. These biosensors are based on immobilization of antibodies on gold nanostructured screen-printed electrodes with cyclic voltammetry detection [115], or with difference pulse voltammetry detection with immobilization on gelsolin coated electrodes that selectively binds A*β* peptides [116]. Another interesting approach combines ELISA and surface plasmon resonance to provide greatly enhanced detection, using gold nano-particles conjugated with antibodies [117]. The role of the nano-particles in this assay is to increase the change in refractive index response that each immobilized molecule produces. This provides detection limits as low as single molecules. This technique is also designed to handle precipitates as part of the detection assay, which may be an advantage when working with oligomerization states. The last group of methods allow direct observation of amyloid plaques in vivo. This direct observation is appealing for clinical use, but is not yet practiced routinely. Methods like magnetic resonance imaging (MRI), positron emission tomography (PET) [118] and diffusion-tensor imaging [119,120] are being developed for direct diagnosis of amyloid plaques based on visual inspection of advanced imaging output. However, all of these methods are based only on qualitative approaches and rely on detecting visible changes in the CNS. There has been some work on quantification of amyloid loads based on PET image analysis but with very limited results [118,121]. More recent advances in MRI are based mainly on the use of particles that allow localization of particular plaques. For example it is possible to use curcumin-conjugated magnetic nano-particles [122] or hollow manganese oxide nano-particles conjugated with a particular antibody [123]. Both of these nano-particle methods increase the specificity and sensitivity of the techniques towards the protein aggregates. However, these approaches are not in routine use and may not satisfy the need for diagnosis before irreparable damage to tissue has occurred.

**Table 2.** Clinical detection of protein aggregates

Proteopathy	Misfolding/oligomerizing protein	Current methods for clinical detection
AD	Aβ peptide	Decrease of marker peptide concentration in CSF, detected by ELISA, immuno-sensors [112,113,115] MRI with plaque selective magnetic nanoparticles – hollow manganese oxide nanoparticles coated with antibody or curcumin-conjugated magnetic nanoparticles [123] Fluorescent labelling of biotic samples with luminescent conjugated oligothiophenes [106,107,109-111] Late phase PET imaging of cerebral fibrillary Aβ peptide with 11C-Pittsburgh compound B as a PET ligand [159,160]
	Tau protein	Ratio of phosphorylated Tau in position 396 and 404 in CSF could discriminate AD from other dementia; Identification by ELISA [161] Identification of phosphorylated biomarkers pTau181, pTau199 and pTau231 in CSF by immunoassays [162-167]
Cerebral amyloid angiopathy	Aβ peptide	Early phase PET imaging of cerebral fibrillary β-amyloid with 11C-Pittsburgh compound B as a PET ligand [168-170]
PD	α-Syn	Detection of α-Syn aggregates in biotic samples by imunohistochemical or fluorescent staining [171-173] Multi-parametric fluorescent pyrene-labelling of biotic samples [174] Detection of α-Syn oligomers in human plasma or red cells by ELISA [175,176]
Huntington’s Disease	Htt	Loss of brain volume observed by MRI with combination of genetic test – identification of abnormal CAG expansion in exon1 of htt gene [177-179]
Variant Creutzfeldt-Jakob disease	Prion protein PrP	Whole blood immunoassay [180-182]
Other prion diseases:	Prion protein PrP	Conformation dependent immunoassays in biopsy samples[183,184]
Gerstmann-Sträussler-Scheinker disease, fatal familial insomnia, kuru, Creutzfeldt-Jakob Disease		
		Analysis of 14-3-3 and PrPSc expression pattern in CSF [185]
		Detection of PrPSc in urine by immunoassay [186,187]

## 5. Concluding remarks and future perspectives

The methods available for detecting proteins associated with neurodegenerative PMDs shows some promise for clinical use. However, many of these methods make no distinction between monomers or oligomers. Thus, the preparation of diagnostic tools that monitor the advancement of oligomerization at an early stage is still under development.

Antibodies are one of the most well established and still promising directions for developing diagnostic tools. Antibodies with ligand conformation sensitivity could be used to build one or more specific standardized clinical ELISA assays for detection of misfolded oligomers. The reliability of such immuno-based approaches is limited by the quality of the antibody involved. Notably, antibody-based detection of the misfolded oligomers, for instance Tau and A *β*, has advanced in recent years (see Table 3a), indicating that standard assays based on immunology may indeed be made sensitive to oligomeric forms. Ensuring that there is a low limit of detection in a complex bio-fluid is also a concern, for ensuring early diagnosis.

Another approach could be the use of native mass spectrometry for detecting protein aggregates. It is a technique which, in contrast to other types of mass spectrometry, can detect non-covalent interactions between proteins [124]**. This technique is also able to detect protein complexes across a wide range of molecular masses and handles heterogeneous samples well. All these features are attractive when aiming to detect oligomers in complicated samples such as bio-fluids. Native mass spectrometry has been used successfully to investigate the assembly of virus capsid, directly from crudely purified culture extract [125]. In principle, it is also possible to detect the oligomers discussed above. An overview of recent, promising use of mass spectroscopy in neurodegenerative PMDs can be found in Table 3c.

The detection of protein glycosylation may be a means for detecting prionic diseases at an early stage (See Table 3b for references). This approach relies upon detailed knowledge of the glycosylation chemistry involved. Although mass spectrometry may be helpful in identifying prion protein glycosylation species, the most promising tool at present are lectins. These are saccharide-binding proteins that can detect differences in glycosylation with some specificity and made a distinction between normal and disease-associated prionic protein successfully [126]. One limit to this approach is the ubiquity of glycosylation; it may not be clear which protein the sugars are actually attached to. For these reasons, false positive results may be a significant problem unless the detection method can also identify the protein involved clearly. Fortunately, many well-stablished antibodies may help solve this problem (Table 3a), although extensive glycosylation may sometimes obscure the epitopes involved.

**Table 3a.** Oligomeric protein states detected by antibodies

Aggregating protein	Antibody	Specificity and epitope	Cross-reactions	Detection Methods and References
Aβ-peptide	4G8	Recognizes residue 18-23 in Aβ sequence, in its fibrils and fibrillary oligomers form	α-Syn, IAPP, Tau These cross-reactions are reported to be fibril-associated, not sequence dependent	WB, IHC, IP, ELISA [188-190]
	A11	Prefibrillar oligomers, not monomers or fibrils	Weakly detects annular oligomeric conformations from α-Syn and IAPP	WB, IHC [189,191]
	6E10	Amino acid 4-9 in Aβ sequence	Detects monomers, oligomers and fibrils, but does not cross-react with α-Syn or IAPP	WB, IHC, IP, ELISA, EM [189,192]
Tau	T22	Human Tau oligomers; conformationally specific epitope	Reported to have no significant cross-reaction with monomers or fibrils of Tau, or with α-Syn, IAPP, or Aβ in any form	WB, IHC, IP ELISA [193-195]
	TOMA	Human Tau oligomers; conformationally specific epitope	No cross-reaction with Tau monomers or fibrils, or with Aβ or α-Syn	WB ,IHC, ELISA [193]
α-Syn	Syn211	Amino acid 121-125 of human α-Syn sequence	Does not cross-react with mouse or rat subtypes. Does not cross-react with β-Syn or β-Syn.	WB, IHC, IP, IF [164]
	Syn-O2	Oligomers, weakly recognizes residue 127-140 of the α-Syn sequence	Does not detect monomers, but detects some fibril	WB, IP, IHC [196,197]
Huntingtin (Htt)	3B5H10	PolyQ in a compact β-strand configuration	Recognizes diseased-associated Htt from human and murine origin; no detectable binding to normal Htt	WB, IHC, IP, ELISA [8,198-200]
	MW1	PolyQ domain of Htt exon 1	Recognizes diseased-associated Htt from human and murine origin; no detectable binding to normal Htt	WB, IHC [200,201]
Prion protein PrPC and PrPSc	6D11	PrpC, human origin; epitope within residues 93-109	Also detects PrPSc. Cross-reacts with prion proteins from cervines, ovine, murine and cricetine	WB, IHC, ELISA, IF [202,203]
	G-12	Amino acid 217-232 human sequence	Murine, human	WB, IP, IF and ELISA [204]
	PRC5	Needs Ala in position 136	PrP from murine, cervines, bovine, ovine, equine, cricetine, mustelines, sciurine, primates	WB [205]
	D18	PrPC, conformationally specific epitope related to Helix 1, residues 130-160	Murine, human, recognises PrPC Only	WB [206-209]
	ICSM18	PrPC, conformationally specific epitope related to Helix 1, residues 130-160	Murine, human, recognises PrPC Only	WB [207-209]
	6H4	PrPSC conformationally specific epitope related to Helix 1, residues 130-160	Also recognises PrPC	WB [207-210]

**Table 3b.** Identification of prion protein glycosylation states

Prion Protein Glycosylation state	Detection method	Sample type	References and notes
Preferential detection of aglycosyl and mono-glycosyl	Antibody PRC7; conformationally specific epitope. Residues at position 154, 166, 185 and 197 are involved.	Extract, WB	The epitope is glycosylation-dependent and residues 154 and 185 are involved [205]
Sialylated and O-glycosidically linked glycans	Lectin proteins affinity for specific glycosylations	Tissue, IHC	Antibodies for PrP (MAB1562 and AB5058) used to ensure that lectins actually detected prion proteins [126]
Glycoproteome of prion protein variants	708 proteins or protein variants assessed	Murine plasma samples	Combined MS-affinity chromatography based approach [211]

**Table 3c.** Identification of oligomeric states by mass spectrometry

Protein	Oligomeric state detected	Sample type	MS detection method sub-type	Reference
α-Syn	Differentiates between oligomers and monomers	Prepared from isolated protein	Hydrogen-Deuterium Exchange, ESI-MS	[212]
α-Syn	Monomers and oligomers	Prepared from isolated protein	ESI-ion mobility mass spectrometry	[213]
α-Syn	Differentiates between oligomers and monomers	Conditioned cell media, similar in complexity to Cerebrospinal Fluid	Combined MS-antibody based approach	[214]
APP, Prion Protein, DJ-1	Monomers and oligomers	Cerebrospinal Fluid	Tandem MS/MS	[215]
Prion Protein	Differentiates between PrPC and PrPSc	Samples prepared from brain homogenate	Quantitative LC–MS/MS	[216]

The problems inherent in the types of detection discussed here—low concentration of target protein, subtle differences between correctly and incorrectly folded and aggregated proteins, heterogeneous protein modifications and strong background signals when detecting in a complex biological environment—do not have obvious solutions. However, it seems likely that protein affinity-based techniques (antibodies, lectins) can successfully be combined with instrument-based detection methods, such as mass-spectrometry, fluorescence, and surface plasmon resonance to produce sensitive detection methods able to identify both the aggregation state and modification state of the protein in question. Moreover, there is reason to believe that early detection can give the patient time to benefit from emerging medical technologies such as antibody-based inhibition of oligomer formation [127].

## List of abbreviations

AD: Alzheimer’s Disease
APP: Amyloid Precursor Protein
Aβ: Beta-Amyloid
BSE: Bovine Spongiform Encephalopathy
CJD: Creutzfeldt-Jakob Disease
CNS: Central Nervous System
IF: Immunofluorescence
IP: Immunoprecipitation
ELISA: Enzyme Linked Immunosorbent Assay
Htt: Huntingtin
HD: Huntington’s Disease
IHC: Immunohistochemistry
LCOs: Luminescent Conjugated Oligothiophenes
PD: Parkinson’s Disease
PrPC: Prion Protein Cellular
PrPSc: Prion Protein Scrapie-associated
PMD: Protein Misfolding Disease
*α*-Syn: *α*-Synuclein
WB: Western Blot

## References

[1] Irwin DJ, Lee VMY, Trojanowski JQ (2013). Parkinson's disease dementia: Convergence of [alpha]-synuclein, tau and amyloid-[beta] pathologies. Nat Rev Neurosci.

[2] Petrou M, Dwamena BA, Foerster BR, MacEachern MP, Bohnen NI, Müller MLTM, Albin RL, Frey KA (2015). Amyloid deposition in parkinson's disease and cognitive impairment: A systematic review. Mov Disord.

[3] Karran E, Mercken M, Strooper BD (2011). The amyloid cascade hypothesis for alzheimer's disease: An appraisal for the development of therapeutics. Nat Rev Drug Discov.

[4] Varadarajan S, Yatin S, Aksenova M, Butterfield DA (2000). Review: Alzheimer's amyloid β-peptide-associated free radical oxidative stress and neurotoxicity. J Struct Biol.

[5] Trevitt CR, Collinge J (2006). A systematic review of prion therapeutics in experimental models. Brain.

[6] Prusiner SB (1998). Prions. Proc Natl Acad Sci U S A.

[7] Morozova OA, Gupta S, Colby DW (2015). Prefibrillar huntingtin oligomers isolated from hd brain potently seed amyloid formation. FEBS Letters.

[8] Owens GE, New DM, West AP, Bjorkman PJ (2015). Anti-polyq antibodies recognize a short polyq stretch in both normal and mutant huntingtin exon 1. J Mol Biol.

[9] Pieri L, Madiona K, Bousset L, Melki R (2012). Fibrillar alpha-synuclein and huntingtin exon 1 assemblies are toxic to the cells. Biophys J.

[10] Kayed R, Sokolov Y, Edmonds B, McIntire TM, Milton SC, Hall JE, Glabe CG (2004). Permeabilization of lipid bilayers is a common conformation-dependent activity of soluble amyloid oligomers in protein misfolding diseases. J Biol Chem.

[11] Selkoe DJ (2001). Alzheimer's disease: Genes, proteins, and therapy. Physiol Rev.

[12] Bros P, Vialaret J, Barthelemy N, Delatour V, Gabelle A, Lehmann S, Hirtz C (2015). Antibody-free quantification of seven tau peptides in human csf using targeted mass spectrometry. Front Neurosci.

[13] Polymeropoulos MH, Lavedan C, Leroy E, Ide SE, Dehejia A, Dutra A, Pike B, Root H, Rubenstein J, Boyer R, Stenroos ES, Chandrasekharappa S, Athanassiadou A, Papapetropoulos T, Johnson WG, Lazzarini AM, Duvoisin RC, Di Iorio G, Golbe LI, Nussbaum RL (1997). Mutation in the α -synuclein gene indentified in families with parkinson's disease. Science.

[14] Spillantini MG, Schmidt ML, Lee VMY, Trojanowski JQ, Jakes R, Goedert M (1997). α-synuclein in lewy bodies. Nature.

[15] Magaki S, Gardner T, Khanlou N, Yong WH, Salmon N, Vinters HV (2015). Brain biopsy in neurologic decline of unknown etiology. Hum Pathol.

[16] Haass C, Steiner H (2001). Protofibrils, the unifying toxic molecule of neurodegenerative disorders?. Nat Neurosci.

[17] Hartmann A, Troadec J-D, Hunot S, Kikly K, Faucheux BA, Mouatt-Prigent A, Ruberg M, Agid Y, Hirsch EC (2001). Caspase-8 is an effector in apoptotic death of dopaminergic neurons in parkinson's disease, but pathway inhibition results in neuronal necrosis. J Neurosci.

[18] Teng L, Zhao J, Wang F, Ma L, Pei G (2010). A gpcr/secretase complex regulates β- and γ-secretase specificity for aβ production and contributes to ad pathogenesis. Cell Res.

[19] Guardia-Laguarta C, Pera M, Lleo A (2010). γ -secretase as a therapeutic target in alzheimers disease. Curr Drug Targets.

[20] Taylor DM (2000). Inactivation of transmissible degenerative encephalopathy agents: A review. Vet J.

[21] Uversky VN, Oldfield CJ, Dunker AK (2008). Intrinsically disordered proteins in human diseases: Introducing the d2 concept. Annu Rev Biophys.

[22] Chiti F, Dobson CM (2006). Protein misfolding, functional amyloid, and human disease. Annu Rev Biochem.

[23] Gregersen N, Bross P, Vang S, Christensen JH (2006). Protein misfolding and human disease. Annual Review of Genomics and Human Genetics.

[24] Naganathan AN, Muñoz V (2005). Scaling of folding times with protein size. J Am Chem Soc.

[25] Juraszek J, Bolhuis PG (2008). Rate constant and reaction coordinate of trp-cage folding in explicit water. Biophys J.

[26] Leal SS, Botelho HM, Gomes CM (2012). Metal ions as modulators of protein conformation and misfolding in neurodegeneration. Coord Chem Rev.

[27] Braselmann E, Chaney JL, Clark PL (2013). Folding the proteome. Trends Biochem Sci.

[28] Yon JM (2002). Protein folding in the post-genomic era. J Cell Mol Med.

[29] Gu Y, Singh A, Bose S, Singh N (2008). Pathogenic mutations in the glycosylphosphatidylinositol signal peptide of prp modulate its topology in neuroblastoma cells. Molecular and Cellular Neuroscience.

[30] Ashok A, Hegde RS (2008). Retrotranslocation of prion proteins from the er by preventing gpi signal sequence transamidation. Mol Biol Cell.

[31] Stelmashook EV, Isaev NK, Genrikhs EE, Amelkina GA, Khaspekov LG, Skrebitsky VG, Illarioshkin SN (2014). Role of zinc and copper ions in the pathogenetic mechanisms of alzheimer’s and parkinson’s diseases. Biochemistry (Moscow).

[32] Nedumpully-Govindan P, Yang Y, Andorfer R, Cao W, Ding F (2015). Promotion or inhibition of islet amyloid polypeptide aggregation by zinc coordination depends on its relative concentration. Biochemistry.

[33] Verasdonck J, Bousset L, Gath J, Melki R, Bockmann A, Meier BH (2016). Further exploration of the conformational space of alpha-synuclein fibrils: Solid-state nmr assignment of a high-ph polymorph. Biomol NMR Assign.

[34] Schmidt M, Sachse C, Richter W, Xu C, Fändrich M, Grigorieff N (2009). Comparison of alzheimer aβ(1–40) and aβ(1–42) amyloid fibrils reveals similar protofilament structures. Proc Natl Acad Sci.

[35] Celej MS, Caarls W, Demchenko AP, Jovin TM (2009). A tripleemission fluorescent probe reveals distinctive amyloid fibrillar polymorphism of wild-type alpha-synuclein and its familial parkinson's disease mutants. Biochemistry.

[36] Bucciantini M, Giannoni E, Chiti F, Baroni F, Formigli L, Zurdo J, Taddei N, Ramponi G, Dobson CM, Stefani M (2002). Inherent toxicity of aggregates implies a common mechanism for protein misfolding diseases. Nature.

[37] Diociaiuti M, Gaudiano MC, Malchiodi-Albedi F (2011). The slowly aggregating salmon calcitonin: A useful tool for the study of the amyloid oligomers structure and activity. Int J Mol Sci.

[38] Lashuel HA, Petre BM, Wall J, Simon M, Nowak RJ, Walz T, Lansbury PT (2002). *α*-synuclein, especially the parkinson's disease-associated mutants, forms pore-like annular and tubular protofibrils. J Mol Biol.

[39] Sokolowski F, Modler AJ, Masuch R, Zirwer D, Baier M, Lutsch G, Moss DA, Gast K, Naumann D (2003). Formation of critical oligomers is a key event during conformational transition of recombinant syrian hamster prion protein. J Biol Chem.

[40] Zakharov VV, Mosevitsky MI (2010). Oligomeric structure of brain abundant proteins gap-43 and basp1. J Struct Biol.

[41] Luth ES, Stavrovskaya IG, Bartels T, Kristal BS, Selkoe DJ (2014). Soluble, prefibrillar alpha-synuclein oligomers promote complex i-dependent, ca2+-induced mitochondrial dysfunction. J Biol Chem.

[42] Walsh P, Vanderlee G, Yau J, Campeau J, Sim VL, Yip CM, Sharpe S (2014). The mechanism of membrane disruption by cytotoxic amyloid oligomers formed by prp(106-126) is dependent on bilayer composition. J Biol Chem.

[43] Kayed R, Pensalfini A, Margol L, Sokolov Y, Sarsoza F, Head E, Hall J, Glabe C (2009). Annular protofibrils are a structurally and functionally distinct type of amyloid oligomer. J Biol Chem.

[44] Ding TT, Lee S-J, Rochet J-C, Lansbury PT (2002). Annular *α*-synuclein protofibrils are produced when spherical protofibrils are incubated in solution or bound to brain-derived membranes†. Biochemistry.

[45] Stöckl M, Fischer P, Wanker E, Herrmann A (2008). *α*-synuclein selectively binds to anionic phospholipids embedded in liquid-disordered domains.. J Mol Biol.

[46] van Rooijen BD, Claessens MMAE, Subramaniam V (2009). Lipid bilayer disruption by oligomeric *α*-synuclein depends on bilayer charge and accessibility of the hydrophobic core. BBA-Biomembranes.

[47] Halskau O, Muga A, Martinez A (2009). Linking new paradigms in protein chemistry to reversible membrane-protein interactions. Curr Protein Pept Sci.

[48] Amtul Z, Uhrig M, Supino R, Beyreuther K (2010). Phospholipids and a phospholipid-rich diet alter the in vitro amyloid-beta peptide levels and amyloid-beta 42/40 ratios. Neurosci Lett.

[49] Gellermann GP, Appel TR, Tannert A, Radestock A, Hortschansky P, Schroeckh V, Leisner C, Lutkepohl T, Shtrasburg S, Rocken C, Pras M, Linke RP, Diekmann S, Fandrich M (2005). Raft lipids as common components of human extracellular amyloid fibrils. Proc Natl Acad Sci U S A.

[50] Benilova I, Karran E, De Strooper B (2012). The toxic aβ oligomer and alzheimer's disease: An emperor in need of clothes. Nat Neurosci.

[51] Saponetti MS, Grimaldi M, Scrima M, Albonetti C, Nori SL, Cucolo A, Bobba F, D'Ursi AM (2014). Aggregation of a β(25-35) on dopc and dopc/dha bilayers: An atomic force microscopy study. Plos One.

[52] Kourie JI, Shorthouse AA (2000). Properties of cytotoxic peptide-formed ion channels. Am J Physiol - Cell Physiol.

[53] Ehrnhoefer DE, Bieschke J, Boeddrich A, Herbst M, Masino L, Lurz R, Engemann S, Pastore A, Wanker EE (2008). Egcg redirects amyloidogenic polypeptides into unstructured, off-pathway oligomers. Nat Struct Mol Biol.

[54] Harroun TA, Bradshaw JP, Ashley RH (2001). Inhibitors can arrest the membrane activity of human islet amyloid polypeptide independently of amyloid formation. FEBS Letters.

[55] Brender JR, Heyl DL, Samisetti S, Kotler SA, Osborne JM, Pesaru RR, Ramamoorthy A (2013). Membrane disordering is not sufficient for membrane permeabilization by islet amyloid polypeptide: Studies of iapp(20-29) fragments. Phys Chem Chem Phys.

[56] Sciacca Michele FM, Milardi D, Messina Grazia ML, Marletta G, Brender Jeffrey R, Ramamoorthy A, La Rosa C (2013). Cations as switches of amyloid-mediated membrane disruption mechanisms: Calcium and iapp. Biophys J.

[57] Sciacca MFM, Brender JR, Lee D-K, Ramamoorthy A (2012). Phosphatidylethanolamine enhances amyloid fiber-dependent membrane fragmentation. Biochemistry.

[58] Hirakura Y, Azimov R, Azimova R, Kagan BL (2000). Polyglutamine-induced ion channels: A possible mechanism for the neurotoxicity of huntington and other cag repeat diseases. J Neuro Sci.

[59] Kagan BL, Jang H, Capone R, Teran Arce F, Ramachandran S, Lal R, Nussinov R (2012). Antimicrobial properties of amyloid peptides. Mol Pharm.

[60] Lin M-C, Mirzabekov T, Kagan BL (1997). Channel formation by a neurotoxic prion protein fragment. J Biol Chem.

[61] Lin H, Bhatia R, Lal R (2001). Amyloid β protein forms ion channels: Implications for alzheimer’s disease pathophysiology. FASEB J.

[62] Hirakura Y, Kagan BL (2001). Pore formation by beta-2-microglobulin: A mechanism for the pathogenesis of dialysis associated amyloidosis. Amyloid.

[63] Hirakura Y, Carreras I, Sipe JD, Kagan BL (2002). Channel formation by serum amyloid a: A potential mechanism for amyloid pathogenesis and host defense. Amyloid.

[64] Demuro A, Mina E, Kayed R, Milton SC, Parker I, Glabe CG (2005). Calcium dysregulation and membrane disruption as a ubiquitous neurotoxic mechanism of soluble amyloid oligomers. J Biol Chem.

[65] Dante S, Hauss T, Brandt A, Dencher NA (2008). Membrane fusogenic activity of the alzheimer's peptide aβ(1-42) demonstrated by small-angle neutron scattering. J Mol Biol.

[66] Sokolov Y, Kozak JA, Kayed R, Chanturiya A, Glabe C, Hall JE (2006). Soluble amyloid oligomers increase bilayer conductance by altering dielectric structure. J Gen Physiol.

[67] Simons K, Ikonen E (1997). Functional rafts in cell membranes. Nature.

[68] Sevcsik E, Brameshuber M, Folser M, Weghuber J, Honigmann A, Schutz GJ (2015). Gpi-anchored proteins do not reside in ordered domains in the live cell plasma membrane. Nat Commun.

[69] Valdes-Gonzalez T, Inagawa J, Ido T (2001). Neuropeptides interact with glycolipid receptors a surface plasmon resonance study. Peptides.

[70] Mahfoud R, Garmy N, Maresca M, Yahi N, Puigserver A, Fantini J (2002). Identification of a common sphingolipid-binding domain in alzheimer, prion, and hiv-1 proteins. J Biol Chem.

[71] Hebbar S, Lee E, Manna M, Steinert S, Kumar GS, Wenk M, Wohland T, Kraut R (2008). A fluorescent sphingolipid binding domain peptide probe interacts with sphingolipids and cholesterol-dependent raft domains. J Lip Res.

[72] Yanagisawa K, Odaka A, Suzuki N, Ihara Y (1995). Gm1 ganglioside-bound amyloid β-protein (aβ): A possible form of preamyloid in alzheimer's disease. Nat Med.

[73] Martinez Z, Zhu M, Han S, Fink AL (2007). Gm1 specifically interacts with *α*-synuclein and inhibits fibrillation†. Biochemistry.

[74] Di Pasquale E, Fantini J, Chahinian H, Maresca M, Taïeb N, Yahi N (2010). Altered ion channel formation by the parkinson's-disease-linked e46k mutant of *α*-synuclein is corrected by gm3 but not by gm1 gangliosides. J Mol Biol.

[75] Mattei V, Garofalo T, Misasi R, Circella A, Manganelli V, Lucania G, Pavan A, Sorice M (2004). Prion protein is a component of the multimolecular signaling complex involved in t cell activation. FEBS Letters.

[76] Sanghera N, Pinheiro TJT (2002). Binding of prion protein to lipid membranes and implications for prion conversion1. J Mol Biol.

[77] Jo E, McLaurin J, Yip CM, St. George-Hyslop P, Fraser PE (2000). *α*-synuclein membrane interactions and lipid specificity. J Biol Chem.

[78] Ramakrishnan M, Jensen PH, Marsh D (2003). *α*-synuclein association with phosphatidylglycerol probed by lipid spin labels†. Biochemistry.

[79] Kubo S-i, Nemani VM, Chalkley RJ, Anthony MD, Hattori N, Mizuno Y, Edwards RH, Fortin DL (2005). A combinatorial code for the interaction of *α*-synuclein with membranes. J Biol Chem.

[80] Pandit SA, Bostick D, Berkowitz ML (2004). Complexation of phosphatidylcholine lipids with cholesterol. Biophys J.

[81] Barenholz Y, Quinn P (2004). Sphingomyelin and cholesterol: From membrane biophysics and rafts to potential medical applications. Membrane dynamics and domains.

[82] Veiga MP, Arrondo JLR, Goñi FM, Alonso A, Marsh D (2001). Interaction of cholesterol with sphingomyelin in mixed membranes containing phosphatidylcholine, studied by spin-label esr and ir spectroscopies. A possible stabilization of gel-phase sphin-golipid domains by cholesterol†. Biochemistry.

[83] Van Echteld CJA, De Kruijff B, Mandersloot JG, De Gier J (1981). Effects of lysophosphatidylcholines on phosphatidylcholine and phosphatidylcholine/cholesterol liposome systems as revealed by 31p-nmr, electron microscopy and permeability studies. BBA-Biomembranes.

[84] Urbina JA, Pekerar S, Le H-b, Patterson J, Montez B, Oldfield E (1995). Molecular order and dynamics of phosphatidylcholine bilayer membranes in the presence of cholesterol, ergosterol and lanosterol: A comparative study using 2h-, 13c- and 31p-nmr spectroscopy. BBA-Biomembranes.

[85] Snyder B, Freire E (1980). Compositional domain structure in phosphatidylcholine-cholesterol and sphingomyelin-cholesterol bilayers. Proc Natl Acad Sci U S A.

[86] Nyberg L, Duan R-D, Nilsson Å (2000). A mutual inhibitory effect on absorption of sphingomyelin and cholesterol. J Nutr Biochem.

[87] McIntosh TJ, Magid AD, Simon SA (1989). Cholesterol modifies the short-range repulsive interactions between phosphatidylcholine membranes. Biochemistry.

[88] Li X-M, Ramakrishnan M, Brockman HL, Brown RE, Swamy MJ (2002). N-myristoylated phosphatidylethanolamine: Interfacial behavior and interaction with cholesterol. Langmuir.

[89] Fantini J, Yahi N (2010). Molecular insights into amyloid regulation by membrane cholesterol and sphingolipids: Common mechanisms in neurodegenerative diseases. Expert Rev Mol Med.

[90] Harris JR (2002). In vitro fibrillogenesis of the amyloid beta 1-42 peptide: Cholesterol potentiation and aspirin inhibition. Micron.

[91] Arispe N, Doh M (2002). Plasma membrane cholesterol controls the cytotoxicity of alzheimer’s disease aβp (1–40) and (1–42) peptides. FASEB J.

[92] Yip CM, Elton EA, Darabie AA, Morrison MR, Cholesterol McLaurin J. (2001). a modulator of membrane-associated aβ-fibrillogenesis and neurotoxicity1. J Mol Biol.

[93] Hartmann T, Kuchenbecker J, Grimm MOW (2007). Alzheimer’s disease: The lipid connection. J Neurochem.

[94] Cole SL, Grudzien A, Manhart IO, Kelly BL, Oakley H, Vassar R (2005). Statins cause intracellular accumulation of amyloid precursor protein, beta-secretase-cleaved fragments, and amyloid beta-peptide via an isoprenoid-dependent mechanism. J Biol Chem.

[95] Bar-On P, Rockenstein E, Adame A, Ho G, Hashimoto M, Masliah E (2006). Effects of the cholesterol-lowering compound methyl-β-cyclodextrin in models of *α*-synucleinopathy. J Neurochem.

[96] Bosco DA, Fowler DM, Zhang Q, Nieva J, Powers ET, Wentworth P, Lerner RA, Kelly JW (2006). Elevated levels of oxidized cholesterol metabolites in lewy body disease brains accelerate α-synuclein fibrilization. Nat Chem Biol.

[97] Chang S, Bray SM, Li Z, Zarnescu DC, He C, Jin P, Warren ST (2008). Identification of small molecules rescuing fragile x syndrome phenotypes in drosophila. Nat Chem Biol.

[98] Hashimoto M, Katakura M, Hossain S, Rahman A, Shimada T, Shido O (2011). Docosahexaenoic acid withstands the abeta(25-35)-induced neurotoxicity in sh-sy5y cells. J Nutr Biochem.

[99] Hashimoto M, Shahdat HM, Yamashita S, Katakura M, Tanabe Y, Fujiwara H, Gamoh S, Miyazawa T, Arai H, Shimada T, Shido O (2008). Docosahexaenoic acid disrupts in vitro amyloid beta(1-40) fibrillation and concomitantly inhibits amyloid levels in cerebral cortex of alzheimer's disease model rats. J Neurochem.

[100] Cole GM, Frautschy SA (2006). Docosahexaenoic acid protects from amyloid and dendritic pathology in an alzheimer's disease mouse model. Nutr Health.

[101] Rescigno T, Capasso A, Tecce MF (2016). Effect of docosahexaenoic acid on cell cycle pathways in breast cell lines with different transformation degree. J Cell Physiol.

[102] Kotarek JA, Moss MA (2010). Impact of phospholipid bilayer saturation on amyloid-beta protein aggregation intermediate growth: A quartz crystal microbalance analysis. Anal Biochem.

[103] Viola KL, Klein WL (2015). Amyloid beta oligomers in alzheimer's disease pathogenesis, treatment, and diagnosis. Acta Neuropathol.

[104] Levine H (1993). Thioflavine-t interaction with synthetic alzheimers-disease beta-amyloid peptides - detection of amyloid aggregation in solution. Prot Sci.

[105] Miller DL, Potempska A, Wegiel J, Mehta PD (2011). High-affinity rabbit monoclonal antibodies specific for amyloid peptides amyloid-beta(40) and amyloid-beta(42). J Alzheimers Dis.

[106] Johansson LBG, Simon R, Bergstrom G, Eriksson M, Prokop S, Mandenius C-F, Heppner FL, Aslund AKO, Nilsson KPR (2015). An azide functionalized oligothiophene ligand - a versatile tool for multimodal detection of disease associated protein aggregates. Biosens Bioelectron.

[107] Nystrom S, Psonka-Antonczyk KM, Ellingsen PG, Johansson LBG, Reitan N, Handrick S, Prokop S, Heppner FL, Wegenast-Braun BM, Jucker M, Lindgren M, Stokke BT, Hammarstrom P, Nilsson KPR (2013). Evidence for age-dependent in vivo conformational rearrangement within a beta amyloid deposits. ACS Chem Biol.

[108] Klingstedt T, Nilsson KPR (2011). Conjugated polymers for enhanced bioimaging. BBA-General Subjects.

[109] Nyström S, Psonka-Antonczyk KM, Ellingsen PG, Johansson LBG, Reitan N, Handrick S, Prokop S, Heppner FL, Wegenast-Braun BM, Jucker M, Lindgren M, Stokke BT, Hammarström P (2013). Nilsson KPR. Evidence for age-dependent in vivo conformational rearrangement within a β amyloid deposits. ACS Chem Biol.

[110] Klingstedt T, Shirani H, Åslund KOA, Cairns NJ, Sigurdson CJ, Goedert M, Nilsson KPR (2013). The structural basis for optimal performance of oligothiophene-based fluorescent amyloid ligands: Conformational flexibility is essential for spectral assignment of a diversity of protein aggregates. Chem-Euro J.

[111] Ren W, Xu M, Liang SH, Xiang H, Tang L, Zhang M, Ding D, Li X, Zhang H, Hu Y (2016). Discovery of a novel fluorescent probe for the sensitive detection of beta-amyloid deposits. Biosens Bioelectron.

[112] Lehallier B, Essioux L, Gayan J (2015). Combined plasma and cerebrospinal fluid signature for the prediction of midterm progression from mild cognitive impairment to alzheimer disease. JAMA Neurol.

[113] David MA, Tayebi M (2014). Detection of protein aggregates in brain and cerebrospinal fluid derived from multiple sclerosis patients. Front Neuro.

[114] Shankar GM, Li S, Mehta TH, Garcia-Munoz A, Shepardson NE, Smith I, Brett FM, Farrell MA, Rowan MJ, Lemere CA, Regan CM, Walsh DM, Sabatini BL, Selkoe DJ (2008). Amyloid-beta protein dimers isolated directly from alzheimer's brains impair synaptic plasticity and memory. Nature Med.

[115] Rama EC, Gonzalez-Garcia MB, Costa-Garcia A (2014). Competitive electrochemical immunosensor for amyloid-beta 1-42 detection based on gold nanostructurated screen-printed carbon electrodes. Sens Actuators B-Chem.

[116] Yu YY, Zhang L, Li CL, Sun XY, Tang DQ, Shi GY (2014). A method for evaluating the level of soluble beta-amyloid((1-40/1-42)) in alzheimer's disease based on the binding of gelsolin to beta-amyloid peptides. Angew Chem Int Ed.

[117] Chen S, Svedendahl M, Antosiewicz TJ, Käll M (2013). Plasmon-enhanced enzyme-linked immunosorbent assay on large arrays of individual particles made by electron beam lithography.. ACS Nano.

[118] Schmidt ME, Chiao P, Klein G, Matthews D, Thurfjell L, Cole PE, Margolin R, Landau S, Foster NL, Mason NS, De Santi S, Suhy J, Koeppe RA, Jagust W, Alzheimer's Dis N (2015). The influence of biological and technical factors on quantitative analysis of amyloid pet: Points to consider and recommendations for controlling variability in longitudinal data. Alzheimers Dem.

[119] Molinuevo JL, Ripolles P, Simo M, Llado A, Olives J, Balasa M, Antonell A, Rodriguez-Fornells A, Rami L (2014). White matter changes in preclinical alzheimer's disease: A magnetic resonance imaging-diffusion tensor imaging study on cognitively normal older people with positive amyloid beta protein 42 levels. Neurobiol Aging.

[120] Kantarci K, Schwarz CG, Reid RI, Przybelski SA, Lesnick TG, Zuk SM, Senjem ML, Gunter JL, Lowe V, Machulda MM, Knopman DS, Petersen RC, Jack CR (2014). White matter integrity determined with diffusion tensor imaging in older adults without dementia influence of amyloid load and neurodegeneration. Jama Neurol.

[121] Soares JM, Marques P, Alves V, Sousa N (2013). A hitchhiker's guide to diffusion tensor imaging. Front Neurosci.

[122] Cheng KK, Chan PS, Fan S, Kwan SM, Yeung KL, Wang Y-XJ, Chow AHL, Wu EX, Baum L (2015). Curcumin-conjugated magnetic nanoparticles for detecting amyloid plaques in alzheimer's disease mice using magnetic resonance imaging (mri). Biomaterials.

[123] Kim JH, Ha TL, Im GH, Yang J, Seo SW, Lee IS, Lee JH (2013). Magnetic resonance imaging of amyloid plaques using hollow manganese oxide nanoparticles conjugated with antibody a beta 1-40 in a transgenic mouse model. Neuroreport.

[124] Erba EB (2014). Investigating macromolecular complexes using top-down mass spectrometry. Proteomics.

[125] Shoemaker GK, van Duijn E, Crawford SE, Uetrecht C, Baclayon M, Roos WH, Wuite GJL, Estes MK, Prasad BVV, Heck AJR (2010). Norwalk virus assembly and stability monitored by mass spectrometry. Mol Cell Proteomics.

[126] Zomosa-Signoret V, Mayoral M, Limon D, Espinosa B, Calvillo M, Zenteno E, Martinez V, Guevara J (2011). Sialylated and o-glycosidically linked glycans in prion protein deposits in a case of gerstmann-straussler-scheinker disease. Neuropathol.

[127] Lambert MP, Velasco PT, Chang L, Viola KL, Fernandez S, Lacor PN, Khuon D, Gong Y, Bigio EH, Shaw P, De Felice FG, Krafft GA, Klein WL (2007). Monoclonal antibodies that target pathological assemblies of abeta. J Neurochem.

[128] Burns A, Iliffe S (2009). Alzheimer’s disease. BMJ.

[130] Wenk GL (2003). Neuropathologic changes in alzheimer's disease. J Clin Psychiatry.

[130] Revesz T, Holton JL, Lashley T, Plant G, Frangione B, Rostagno A, Ghiso J (2009). Genetics and molecular pathogenesis of sporadic and hereditary cerebral amyloid angiopathies. Acta Neuropathol.

[131] Kalia LV, Lang AE (2015). Parkinson's disease. Lancet.

[132] Lei P, Ayton S, Finkelstein DI, Adlard PA, Masters CL, Bush AI (2010). Tau protein: Relevance to parkinson's disease. Int J Biochem Cell Biol.

[133] Bang J, Spina S, Miller BL (2015). Frontotemporal dementia. Lancet.

[134] van der Zee J, Van Broeckhoven C (2014). Dementia in 2013: Frontotemporal lobar degeneration-building on breakthroughs. Nat Rev Neurol.

[135] Dayalu P, Albin RL (2015). Huntington disease: Pathogenesis and treatment. Neurol Clin.

[136] Garringer HJ, Murrell J, D'Adamio L, Ghetti B, Vidal R (2010). Modeling familial british and danish dementia. Brain Struct Funct.

[137] Rostagno A, Tomidokoro Y, Lashley T, Ng D, Plant G, Holton J, Frangione B, Revesz T, Ghiso J (2005). Chromosome 13 dementias. Cell Mol Life Sci.

[138] Herve D, Chabriat H (2010). Cadasil. J Geriatr Psychiatry Neurol.

[139] Viswanathan A, Gschwendtner A, Guichard JP, Buffon F, Cumurciuc R, O'Sullivan M, Holtmannspotter M, Pachai C, Bousser MG, Dichgans M, Chabriat H (2007). Lacunar lesions are independently associated with disability and cognitive impairment in cadasil. Neurology.

[140] Quinlan RA, Brenner M, Goldman JE, Messing A (2007). Gfap and its role in alexander disease. Exp Cell Res.

[141] Yoshida T, Nakagawa M (2012). Clinical aspects and pathology of alexander disease, and morphological and functional alteration of astrocytes induced by gfap mutation. Neuropathol.

[142] Miranda E, Lomas DA (2006). Neuroserpin: A serpin to think about. Cell Mol Life Sci.

[143] Davis RL, Shrimpton AE, Holohan PD, Bradshaw C, Feiglin D, Collins GH, Sonderegger P, Kinter J, Becker LM, Lacbawan F, Krasnewich D, Muenke M, Lawrence DA, Yerby MS, Shaw CM, Gooptu B, Elliott PR, Finch JT, Carrell RW, Lomas DA (1999). Familial dementia caused by polymerization of mutant neuroserpin. Nature.

[144] Imran M, Mahmood S (2011). An overview of human prion diseases. Virol J.

[145] Budka H (2003). Neuropathology of prion diseases. Br Med Bull.

[146] Takada LT, Geschwind MD (2013). Prion diseases. Semin Neurol.

[147] De Michele G, Pocchiari M, Petraroli R, Manfredi M, Caneve G, Coppola G, Casali C, Sacca F, Piccardo P, Salvatore E, Berardelli A, Orio M, Barbieri F, Ghetti B, Filla A (2003). Variable phenotype in a p102l gerstmann-straussler-scheinker italian family. Can J Neurol Sci.

[148] Tateishi J, Brown P, Kitamoto T, Hoque ZM, Roos R, Wollman R, Cervenakova L, Gajdusek DC (1995). First experimental transmission of fatal familial insomnia. Nature.

[149] Dickson DW, Rademakers R, Hutton ML (2007). Progressive supranuclear palsy: Pathology and genetics. Brain Pathol.

[150] Ling H (2016). Clinical approach to progressive supranuclear palsy. J Mov Disord.

[151] Baugh CM, Stamm JM, Riley DO, Gavett BE, Shenton ME, Lin A, Nowinski CJ, Cantu RC, McKee AC, Stern RA (2012). Chronic traumatic encephalopathy: Neurodegeneration following repetitive concussive and subconcussive brain trauma. Brain Imaging Behav.

[152] Stein TD, Alvarez VE, McKee AC (2014). Chronic traumatic encephalopathy: A spectrum of neuropathological changes following repetitive brain trauma in athletes and military personnel. Alzheimers Res Ther.

[153] Steele JC (2005). Parkinsonism-dementia complex of guam. Mov Disord.

[154] Halper J, Scheithauer BW, Okazaki H, Laws ER (1986). Meningio-angiomatosis: A report of six cases with special reference to the occurrence of neurofibrillary tangles. J Neuropathol Exp Neurol.

[155] Wiebe S, Munoz DG, Smith S, Lee DH (1999). Meningioangiomatosis. A comprehensive analysis of clinical and laboratory features. Brain.

[156] Bennett MJ, Rakheja D (2013). The neuronal ceroid-lipofuscinoses. Dev Disabil Res Rev.

[157] Vesa J, Hellsten E, Verkruyse LA, Camp LA, Rapola J, Santavuori P, Hofmann SL, Peltonen L (1995). Mutations in the palmitoyl protein thioesterase gene causing infantile neuronal ceroid lipofuscinosis. Nature.

[158] Ferrer I, Santpere G, van Leeuwen FW (2008). Argyrophilic grain disease. Brain.

[159] Klunk WE, Engler H, Nordberg A, Wang YM, Blomqvist G, Holt DP, Bergstrom M, Savitcheva I, Huang GF, Estrada S, Ausen B, Debnath ML, Barletta J, Price JC, Sandell J, Lopresti BJ, Wall A, Koivisto P, Antoni G, Mathis CA, Langstrom B (2004). Imaging brain amyloid in alzheimer's disease with pittsburgh compound-b. Ann Neurol.

[160] Marcus C, Mena E, Subramaniam RM (2014). Brain pet in the diagnosis of alzheimer's disease. Clin Nucl Med.

[161] Hu YY, He SS, Wang XC, Duan QH, Grundke-Iqbal I, Iqbal K, Wang JZ (2002). Levels of nonphosphorylated and phosphorylated tau in cerebrospinal fluid of alzheimer's disease patients - an ultrasensitive bienzyme-substrate-recycle enzyme-linked immunosorbent assay. Am J Pathol.

[162] Lewczuk P, Esselmann H, Bibl M, Beck G, Maler JM, Otto M, Kornhuber J, Wiltfang L (2004). Tau protein phosphorylated at threonine 181 in csf as a neurochemical biomarker in alzheimer's disease-original data and review of the literature. J Mol Neurosci.

[163] Lewczuk P, Kornhuber J (2011). Neurochemical dementia diagnostics in alzheimer's disease: Where are we now and where are we going?. Expert Rev Proteomics.

[164] Parnetti L, Chiasserini D, Persichetti E, Eusebi P, Varghese S, Qureshi MM, Dardis A, Deganuto M, De Carlo C, Castrioto A, Balducci C, Paciotti S, Tambasco N, Bembi B, Bonanni L, Onofrj M, Rossi A, Beccari T, El-Agnaf O, Calabresi P (2014). Cerebrospinal fluid lysosomal enzymes and alpha-synuclein in parkinson's disease. Mov Disord.

[165] Itoh N, Arai H, Urakami K, Ishiguro K, Ohno H, Hampel H, Buerger K, Wiltfang J, Otto M, Kretzschmar H, Moeller HJ, Imagawa M, Kohno H, Nakashima K, Kuzuhara S, Sasaki H, Imahori K (2001). Large-scale, multicenter study of cerebrospinal fluid tau protein phosphorylated at serine 199 for the antemortem diagnosis of alzheimer's disease. Ann Neurol.

[166] Hampel H, Buerger K, Kohnken R, Teipel SJ, Zinkowski R, Moeller HJ, Rapoport SI, Davies P (2001). Tracking of alzheimer's disease progression with cerebrospinal fluid tau protein phosphorylated at threonine 231. Ann Neurol.

[167] Arai H, Ishiguro K, Ohno H, Moriyama M, Itoh N, Okamura N, Matsui T, Morikawa Y, Horikawa E, Kohno H, Sasaki H, Imahori K (2000). Csf phosphorylated tau protein and mild cognitive impairment: A prospective study. Exp Neurol.

[168] Farid K, Hong YT, Aigbirhio FI, Fryer TD, Menon DK, Warburton EA, Baron JC (2015). Early-phase c-11-pib pet in amyloid angiopathy-related symptomatic cerebral hemorrhage: Potential diagnostic value?. Plos One.

[169] Johnson KA, Gregas M, Becker JA, Kinnecom C, Salat DH, Moran EK, Smith EE, Rosand J, Rentz DM, Klunk WE, Mathis CA, Price JC, DeKosky ST, Fischman AJ, Greenberg SM (2007). Imaging of amyloid burden and distribution in cerebral amyloid angiopathy. Ann Neurol.

[170] Greenberg SM, Grabowski T, Gurol ME, Skehan ME, Nandigam RNK, Becker JA, Garcia-Alloza M, Prada C, Frosch MP, Rosand J, Viswanathan A, Smith EE, Johnson KA (2008). Detection of isolated cerebrovascular beta-amyloid with pittsburgh compound b. Ann Neurol.

[171] Kuusisto E, Parkkinen L, Alafuzoff I (2003). Morphogenesis of lewy bodies: Dissimilar incorporation of alpha-synuclein, ubiquitin, and p62. J Neuropathol Exp Neurol.

[172] Huebinger S, Bannach O, Funke SA, Willbold D, Birkmann E (2012). Detection of alpha-synuclein aggregates by fluorescence microscopy. Rejuvenation Res.

[173] Cook NP, Kilpatrick K, Segatori L, Marti AA (2012). Detection of alpha-synuclein amyloidogenic aggregates in vitro and in cells using light-switching dipyridophenazine ruthenium(ii) complexes. J Am Chem Soc.

[174] Thirunavukkuarasu S, Jares-Erijman EA, Jovin TM (2008). Multiparametric fluorescence detection of early stages in the amyloid protein aggregation of pyrene-labeled alpha-synuclein. J Mol Biol.

[175] El-Agnaf OMA, Salem SA, Paleologou KE, Curran MD, Gibson MJ, Court JA, Schlossmacher MG, Allsop D (2006). Detection of oligomeric forms of alpha-synuclein protein in human plasma as a potential biomarker for parkinson's disease. FASEB J.

[176] Wang XM, Yu S, Li FF, Feng T (2015). Detection of alpha-synuclein oligomers in red blood cells as a potential biomarker of parkinson's disease. Neurosci Letters.

[177] Kassubek J, Juengling FD, Kioschies T, Henkel K, Karitzky J, Kramer B, Ecker D, Andrich J, Saft C, Kraus P, Aschoff AJ, Ludolph AC, Landwehrmeyer GB (2004). Topography of cerebral atrophy in early huntington's disease: A voxel based morphometric mri study. J Neurol Neurosurg Psychiatry.

[178] O'Keeffe GC, Michell AW, Barker RA (2009). Biomarkers in huntington's and parkinson's disease. Biomarkers in Brain Disease.

[179] Ridha BH, Anderson VM, Barnes J, Boyes RG, Price SL, Rossor MN, Whitwell JL, Jenkins L, Black RS, Grundman M, Fox NC (2008). Volumetric mri and cognitive measures in alzheimer disease - comparison of markers of progression. J Neurol.

[180] Edgeworth JA, Farmer M, Sicilia A, Tavares P, Beck J, Campbell T, Lowe J, Mead S, Rudge P, Collinge J, Jackson GS (2011). Detection of prion infection in variant creutzfeldt-jakob disease: A blood-based assay. Lancet.

[181] Sawyer EB, Edgeworth JA, Thomas C, Collinge J, Jackson GS (2015). Preclinical detection of infectivity and disease-specific prp in blood throughout the incubation period of prion disease. Sci Rep.

[182] Lacroux C, Comoy E, Moudjou M, Perret-Liaudet A, Lugan S, Litaise C, Simmons H, Jas-Duval C, Lantier I, Beringue V, Groschup M, Fichet G, Costes P, Streichenberger N, Lantier F, Deslys JP, Vilette D, Andreoletti O (2014). Preclinical detection of variant cjd and bse prions in blood. Plos Pathogens.

[183] Safar JG, Geschwind MD, Deering C, Didorenko S, Sattavat M, Sanchez H, Serban A, Vey M, Baron H, Giles K, Miller BL, DeArmond SJ, Prusiner SB (2005). Diagnosis of human prion disease. Proc Natl Acad Sci U S A.

[184] Nicholson EM (2015). Detection of the disease-associated form of the prion protein in biological samples. Bioanalysis.

[185] Torres M, Cartier L, Matamala JM, Hernandez N, Woehlbier U, Hetz C (2012). Altered prion protein expression pattern in csf as a biomarker for creutzfeldt-jakob disease. Plos One.

[186] Furukawa H, Dohura K, Okuwaki R, Shirabe S, Yamamoto K, Udono H, Ito T, Katamine S, Niwa M (2004). A pitfall in diagnosis of human prion diseases using detection of protease-resistant prion protein in urine - contamination with bacterial outer membrane proteins. J Biol Chem.

[187] Notari S, Qing LT, Pocchiari M, Dagdanova A, Hatcher K, Dogterom A, Groisman JF, Lumholtz IB, Puopolo M, Lasmezas C, Chen SG, Kong QZ, Gambetti P (2012). Assessing prion infectivity of human urine in sporadic creutzfeldt-jakob disease. Emerg Infect Dis.

[188] Oddo S, Billings L, Kesslak JP, Cribbs DH, LaFerla FM (2004). Abeta immunotherapy leads to clearance of early, but not late, hyperphosphorylated tau aggregates via the proteasome. Neuron.

[189] Kayed R, Head E, Sarsoza F, Saing T, Cotman CW, Necula M, Margol L, Wu J, Breydo L, Thompson JL, Rasool S, Gurlo T, Butler P, Glabe CG (2007). Fibril specific, conformation dependent antibodies recognize a generic epitope common to amyloid fibrils and fibrillar oligomers that is absent in prefibrillar oligomers. Mol Neurodegener.

[190] Hatami A, Monjazeb S, Glabe C (2015). The anti-amyloid-beta monoclonal antibody 4g8 recognizes a generic sequence-independent epitope associated with alpha-synuclein and islet amyloid polypeptide amyloid fibrils. J Alzheimers Dis.

[191] Hatami A, Albay R, Monjazeb S, Milton S, Glabe C (2014). Monoclonal antibodies against abeta42 fibrils distinguish multiple aggregation state polymorphisms in vitro and in alzheimer disease brain. J Biol Chem.

[192] Gowert NS, Donner L, Chatterjee M, Eisele YS, Towhid ST, Munzer P, Walker B, Ogorek I, Borst O, Grandoch M, Schaller M, Fischer JW, Gawaz M, Weggen S, Lang F, Jucker M, Elvers M (2014). Blood platelets in the progression of alzheimer's disease. PLoS One.

[193] Castillo-Carranza DL, Sengupta U, Guerrero-Munoz MJ, Lasagna-Reeves CA, Gerson JE, Singh G, Estes DM, Barrett AD, Dineley KT, Jackson GR, Kayed R (2014). Passive immunization with tau oligomer monoclonal antibody reverses tauopathy phenotypes without affecting hyperphosphorylated neurofibrillary tangles. J Neurosci.

[194] Lasagna-Reeves CA, Castillo-Carranza DL, Sengupta U, Sarmiento J, Troncoso J, Jackson GR, Kayed R (2012). Identification of oligomers at early stages of tau aggregation in alzheimer's disease. FASEB J.

[195] Wu JW, Herman M, Liu L, Simoes S, Acker CM, Figueroa H, Steinberg JI, Margittai M, Kayed R, Zurzolo C, Di Paolo G, Duff KE (2013). Small misfolded tau species are internalized via bulk endocytosis and anterogradely and retrogradely transported in neurons. J Biol Chem.

[196] Majbour NK, Vaikath NN, van Dijk KD, Ardah MT, Varghese S, Vesterager LB, Montezinho LP, Poole S, Safieh-Garabedian B, Tokuda T, Teunissen CE, Berendse HW, van de Berg WD, El-Agnaf OM (2016). Oligomeric and phosphorylated alpha-synuclein as potential csf biomarkers for parkinson's disease. Mol Neurodegener.

[197] Vaikath NN, Majbour NK, Paleologou KE, Ardah MT, van Dam E, van de Berg WD, Forrest SL, Parkkinen L, Gai WP, Hattori N, Takanashi M, Lee SJ, Mann DM, Imai Y, Halliday GM, Li JY, El-Agnaf OM (2015). Generation and characterization of novel conformation-specific monoclonal antibodies for alpha-synuclein pathology. Neurobiol Dis.

[198] Peters-Libeu C, Miller J, Rutenber E, Newhouse Y, Krishnan P, Cheung K, Hatters D, Brooks E, Widjaja K, Tran T, Mitra S, Arrasate M, Mosquera LA, Taylor D, Weisgraber KH, Finkbeiner S (2012). Disease-associated polyglutamine stretches in monomeric huntingtin adopt a compact structure. J Mol Biol.

[199] Nucifora LG, Burke KA, Feng X, Arbez N, Zhu S, Miller J, Yang G, Ratovitski T, Delannoy M, Muchowski PJ, Finkbeiner S, Legleiter J, Ross CA, Poirier MA (2012). Identification of novel potentially toxic oligomers formed in vitro from mammalian-derived expanded huntingtin exon-1 protein. J Biol Chem.

[200] Legleiter J, Lotz GP, Miller J, Ko J, Ng C, Williams GL, Finkbeiner S, Patterson PH, Muchowski PJ (2009). Monoclonal antibodies recognize distinct conformational epitopes formed by polyglutamine in a mutant huntingtin fragment. J Biol Chem.

[201] Ko J, Ou S, Patterson PH (2001). New anti-huntingtin monoclonal antibodies: Implications for huntingtin conformation and its binding proteins. Brain Res Bull.

[202] Shiga Y, Miyazawa K, Sato S, Fukushima R, Shibuya S, Sato Y, Konno H, Doh-ura K, Mugikura S, Tamura H, Higano S, Takahashi S, Itoyama Y (2004). Diffusion-weighted mri abnormalities as an early diagnostic marker for creutzfeldt-jakob disease. Neurology.

[203] Wang J, Zhang BY, Zhang J, Xiao K, Chen LN, Wang H, Sun J, Shi Q, Dong XP (2015). Treatment of smb-s15 cells with resveratrol efficiently removes the prp accumulation in vitro and prion infectivity in vivo. Mol Neurobiol.

[204] Caughey B, Raymond GJ, Priola SA, Kocisko DA, Race RE, Bessen RA, Lansbury PT, Chesebro B (1999). Methods for studying prion protein (prp) metabolism and the formation of protease-resistant prp in cell culture and cell-free systems. An update. Mol Biotechnol.

[205] Kang HE, Weng CC, Saijo E, Saylor V, Bian J, Kim S, Ramos L, Angers R, Langenfeld K, Khaychuk V, Calvi C, Bartz J, Hunter N, Telling GC (2012). Characterization of conformation-dependent prion protein epitopes. J Biol Chem.

[206] Beringue V, Vilette D, Mallinson G, Archer F, Kaisar M, Tayebi M, Jackson GS, Clarke AR, Laude H, Collinge J, Hawke S (2004). Prpsc binding antibodies are potent inhibitors of prion replication in cell lines. J Biol Chem.

[207] Doolan KM, Colby DW (2015). Conformation-dependent epitopes recognized by prion protein antibodies probed using mutational scanning and deep sequencing. J Mol Biol.

[208] Enari M, Flechsig E, Weissmann C (2001). Scrapie prion protein accumulation by scrapie-infected neuroblastoma cells abrogated by exposure to a prion protein antibody. Proc Natl Acad Sci U S A.

[209] Peretz D, Williamson RA, Kaneko K, Vergara J, Leclerc E, Schmitt-Ulms G, Mehlhorn IR, Legname G, Wormald MR, Rudd PM, Dwek RA, Burton DR, Prusiner SB (2001). Antibodies inhibit prion propagation and clear cell cultures of prion infectivity. Nature.

[210] Wiseman FK, Cancellotti E, Piccardo P, Iremonger K, Boyle A, Brown D, Ironside JW, Manson JC, Diack AB (2015). The glycosylation status of prpc is a key factor in determining transmissible spongiform encephalopathy transmission between species. J Virol.

[211] Wei X, Herbst A, Ma D, Aiken J, Li L (2011). A quantitative proteomic approach to prion disease biomarker research: Delving into the glycoproteome. J Proteome Res.

[212] Mysling S, Betzer C, Jensen PH, Jorgensen TJ (2013). Characterizing the dynamics of alpha-synuclein oligomers using hydrogen/deuterium exchange monitored by mass spectrometry. Biochemistry.

[213] Illes-Toth E, Ramos MR, Cappai R, Dalton C, Smith DP (2015). Distinct higher-order alpha-synuclein oligomers induce intracellular aggregation. Biochem J.

[214] Emmanouilidou E, Melachroinou K, Roumeliotis T, Garbis SD, Ntzouni M, Margaritis LH, Stefanis L, Vekrellis K (2010). Cell-produced alpha-synuclein is secreted in a calcium-dependent manner by exosomes and impacts neuronal survival. J Neurosci.

[215] Chiasserini D, van Weering JR, Piersma SR, Pham TV, Malekzadeh A, Teunissen CE, de Wit H, Jimenez CR (2014). Proteomic analysis of cerebrospinal fluid extracellular vesicles: A comprehensive dataset. J Proteomics.

[216] Silva CJ, Erickson-Beltran ML, Dynin IC (2016). Covalent surface modification of prions: A mass spectrometry-based means of detecting distinctive structural features of prion strains. Biochemistry.

